# Supplementary material for: Inhibition of protein or glutamine biosynthesis affect the light-induced dephosphorylation of the SBiP1 chaperone in Symbiodiniaceae
Source: Biosci Rep. 2025 Jun 5;45(6):BSR20241085. doi: 10.1042/BSR20241085 (PMC12203957; doi:10.1042/BSR20241085)
Supplement: Online supplementary figure S1 [file BSR-45-06-BSR20241085-s001.pdf]

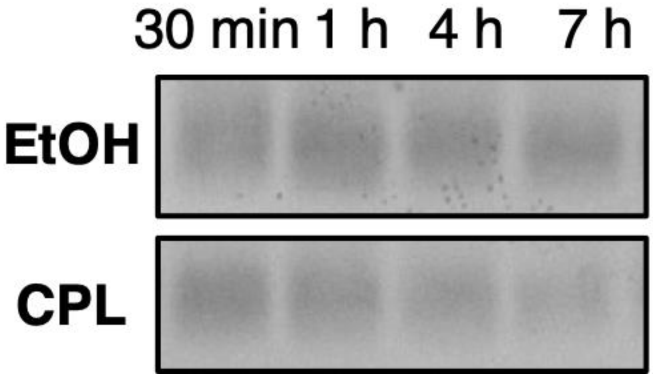

Supplementary Figure 1. Effect of chloramphenicol on D1 protein synthesis. Western blot analysis of D1 protein from CassKB8 cells incubated with 0.1 mM chloramphenicol (CPL) or vehicle (EtOH) for 30 min, 1, 4 nd 7 h. The band intensity of D1 clearly diminished after the CPL treatment (CPL; lanes 30 min, 1-7 h), whereas it remained constant along the same time points when vehicle alone was used for the treatment (EtOH; lanes 30 min, 1-7 h).
