# Supplementary material for: Inhibition of protein or glutamine biosynthesis affect the light-induced dephosphorylation of the SBiP1 chaperone in Symbiodiniaceae
Source: Biosci Rep. 2025 Jun 5;45(6):BSR20241085. doi: 10.1042/BSR20241085 (PMC12203957; doi:10.1042/BSR20241085)

Membranes from experimental replicates for Figure 1

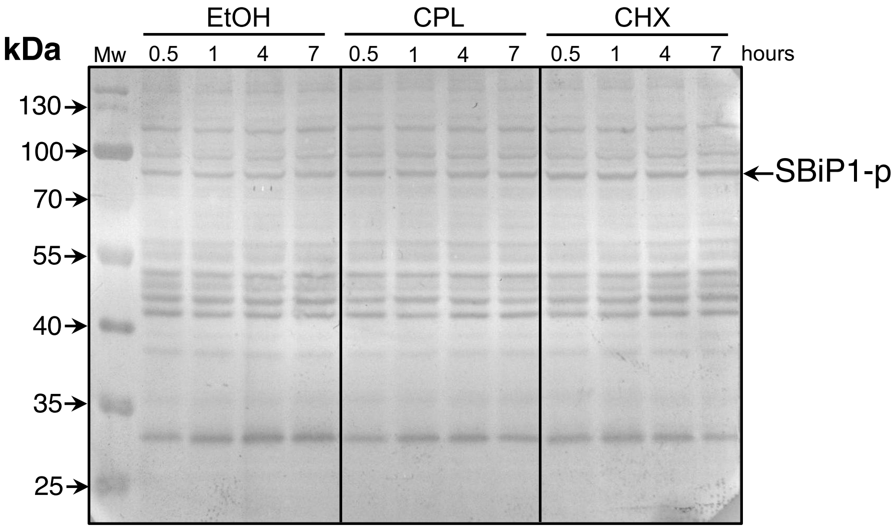

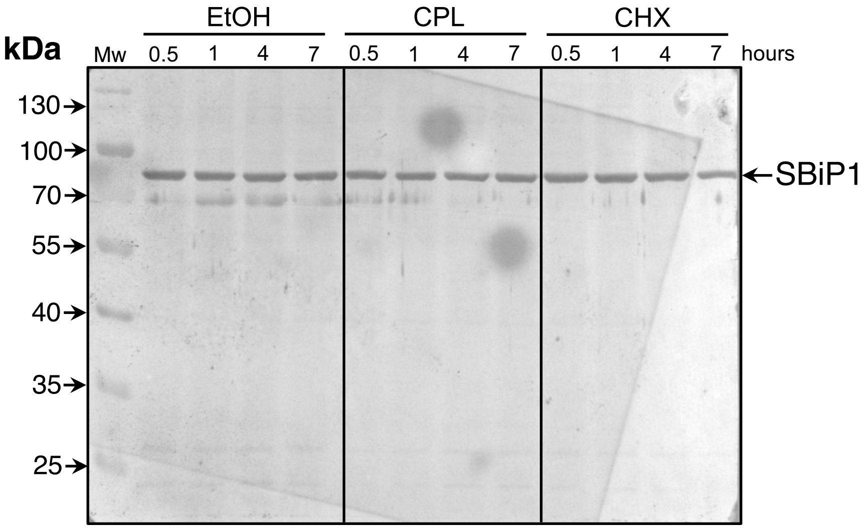

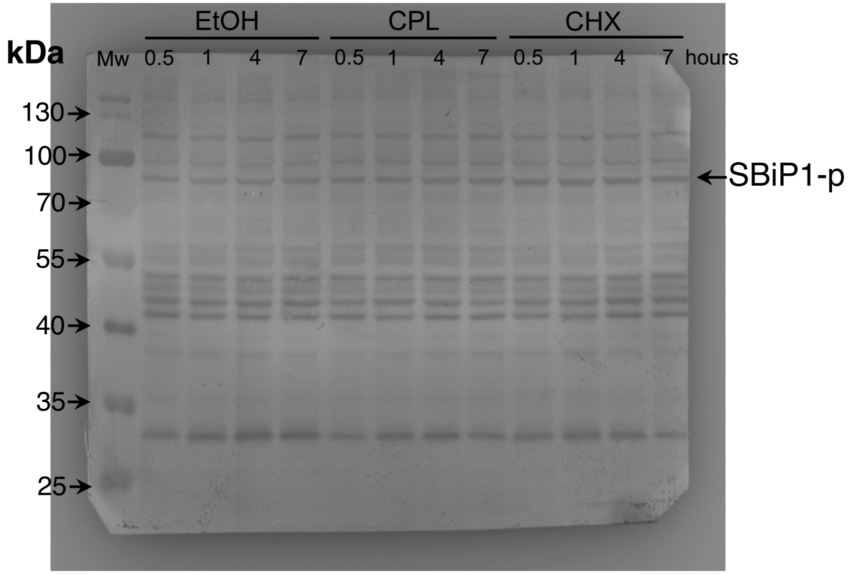

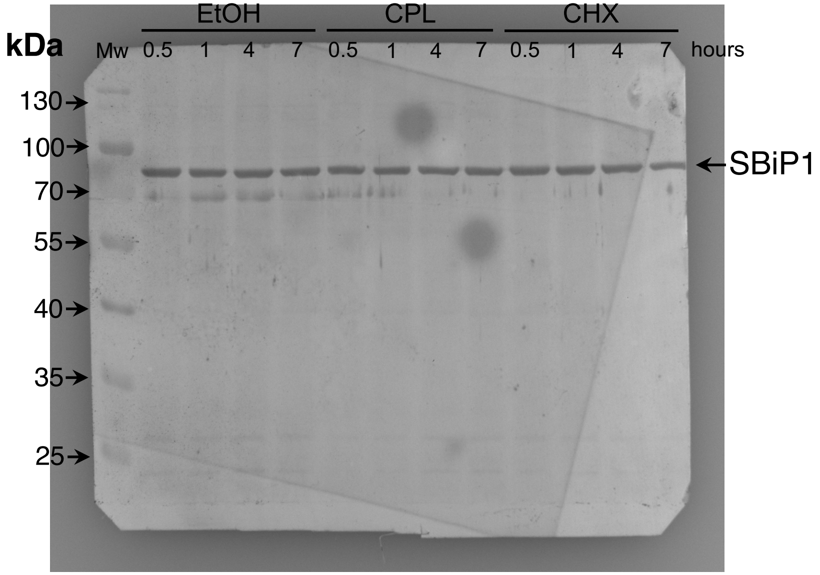

Experimental replicate 1

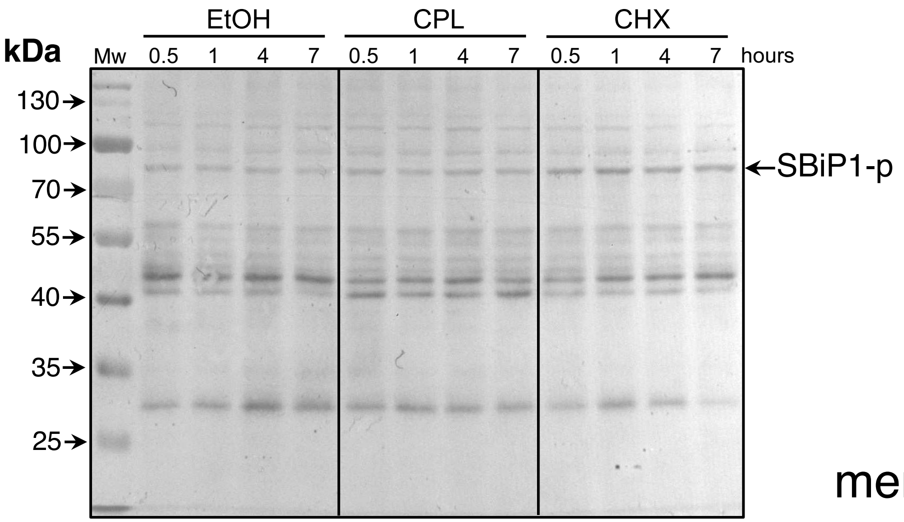

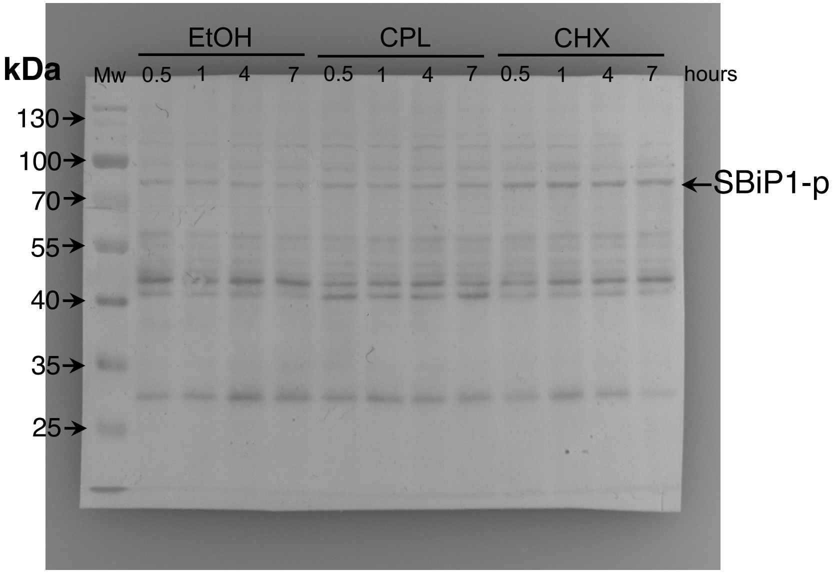

These two membranes were used for Figure 1

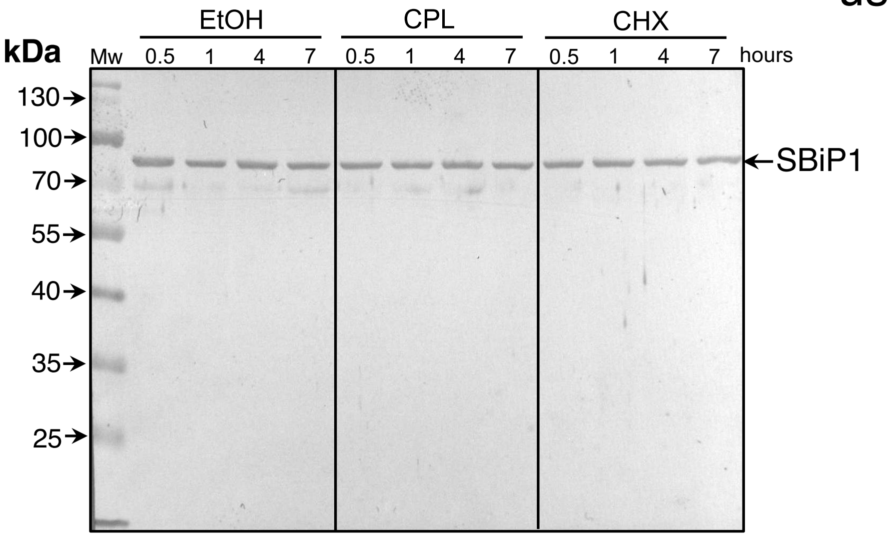

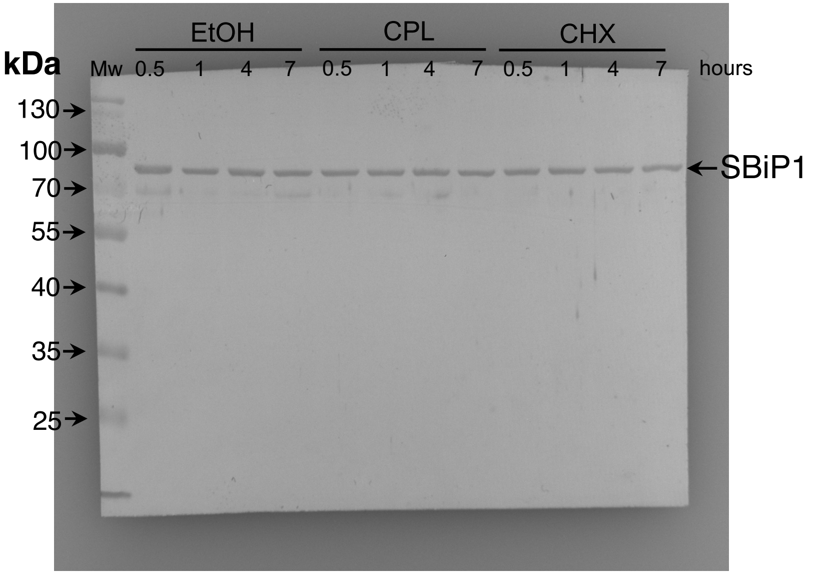

Experimental replicate 2

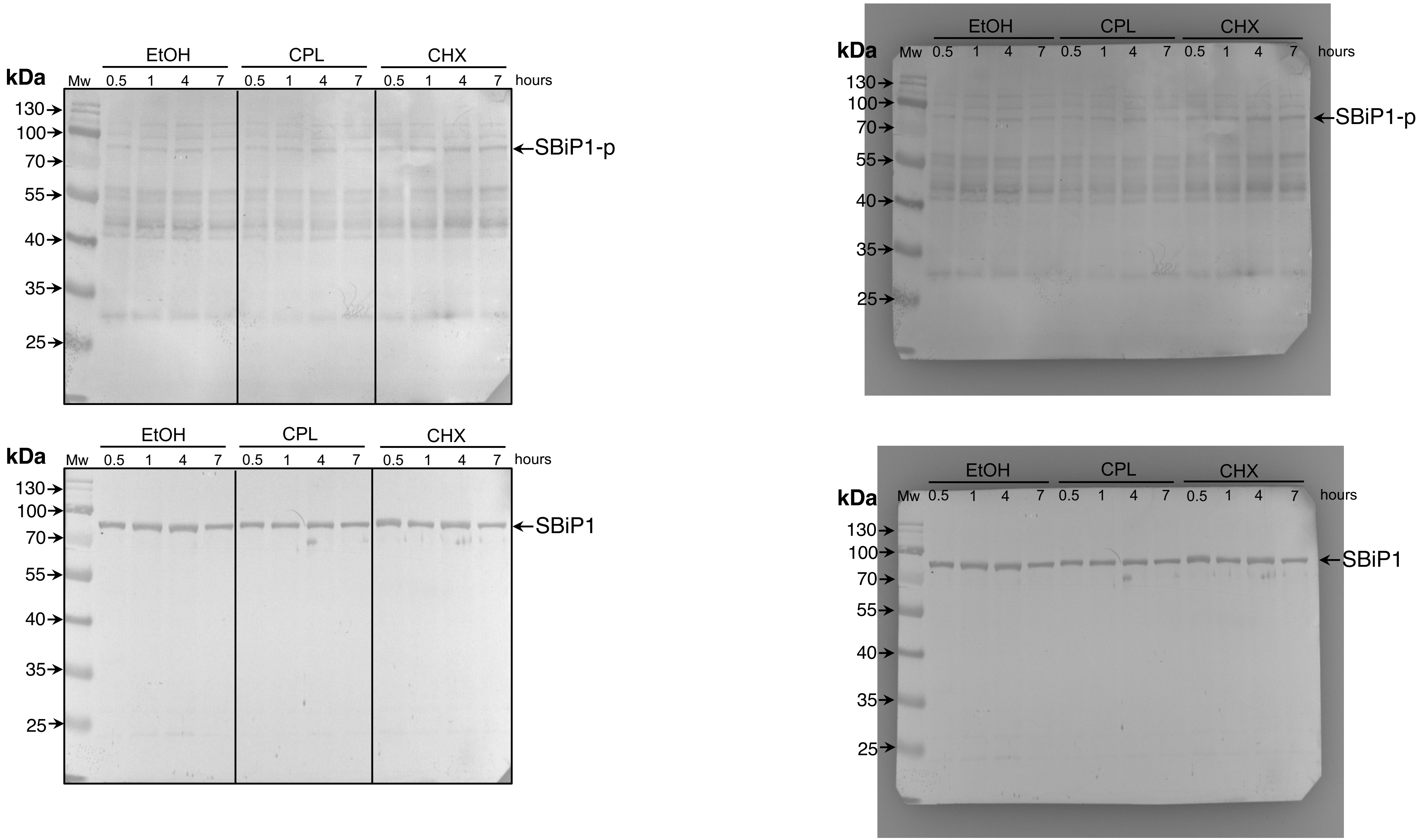

Experimental replicate 3

Membranes from experimental replicates for Figure 2

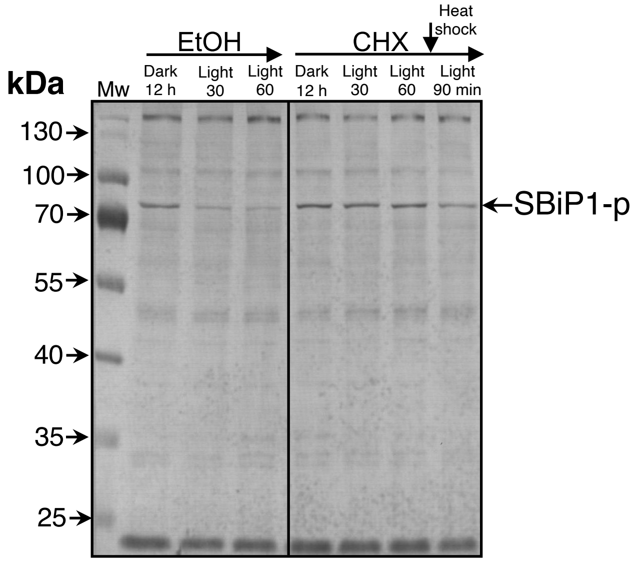

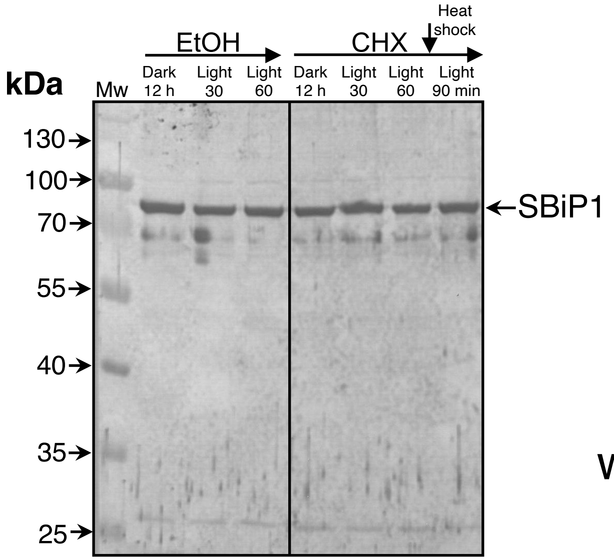

These membranes were used for Figure 2

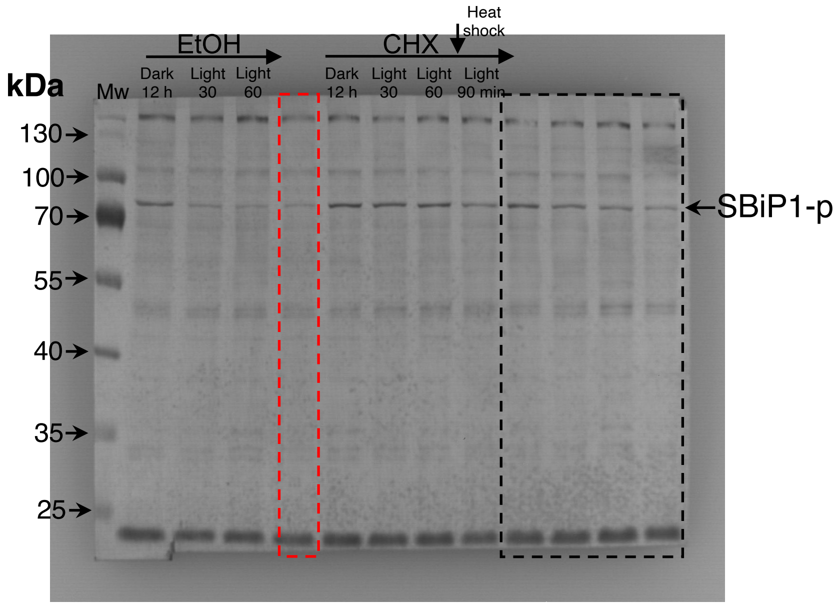

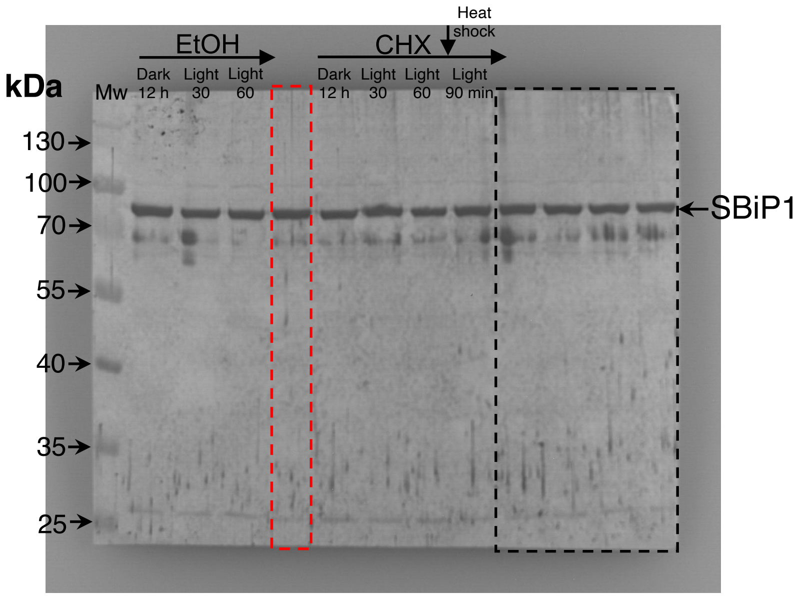

Not used for the figure

Replicate 1

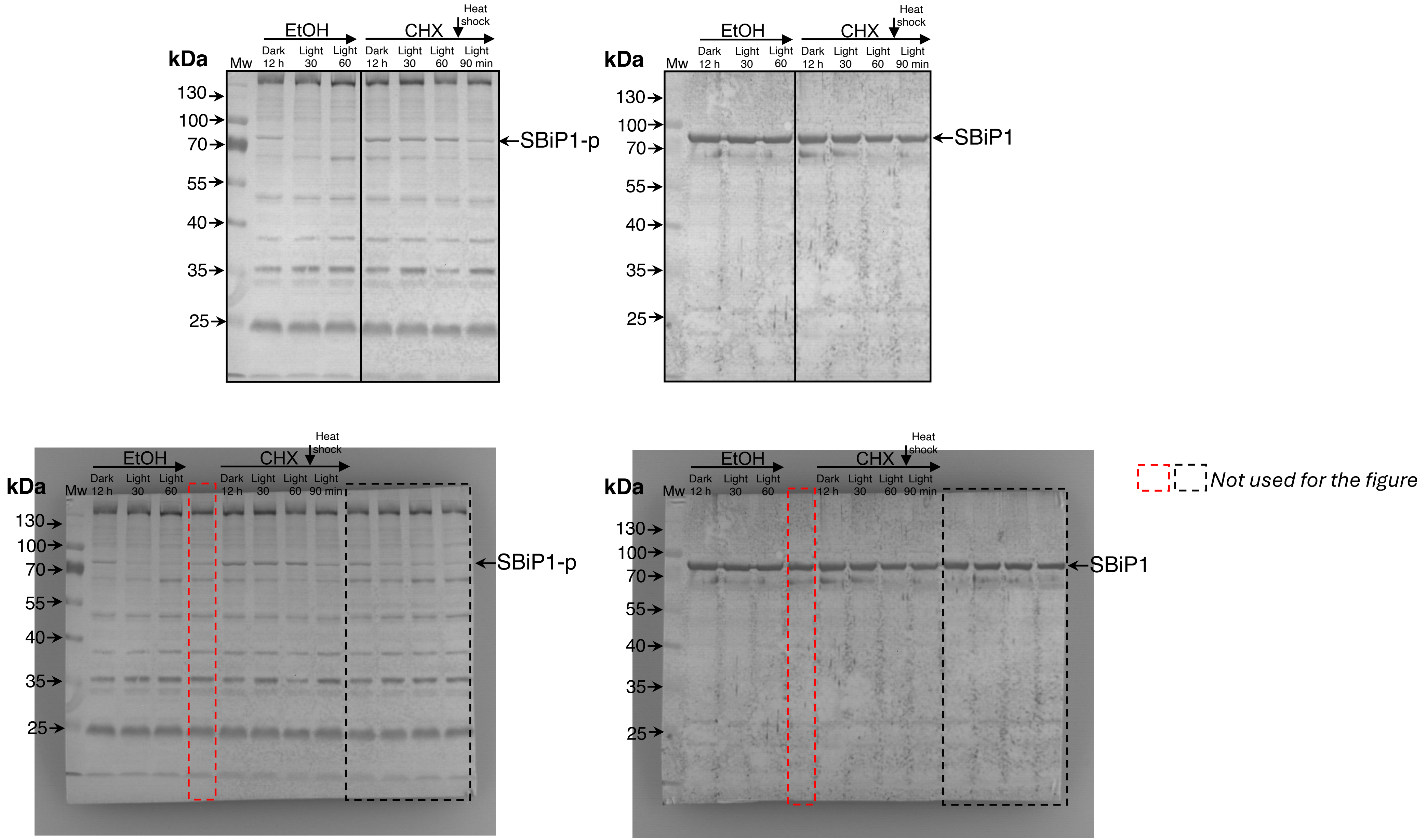
kDa
Mw
EtOH
CHX
Heat shock
Dark 12 h
Light 30
Light 60
Dark 12 h
Light 30
Light 60
Light 90 min
130
100
70
55
40
35
25
SBiP1-p
SBiP1
Not used for the figure

Replicate 2

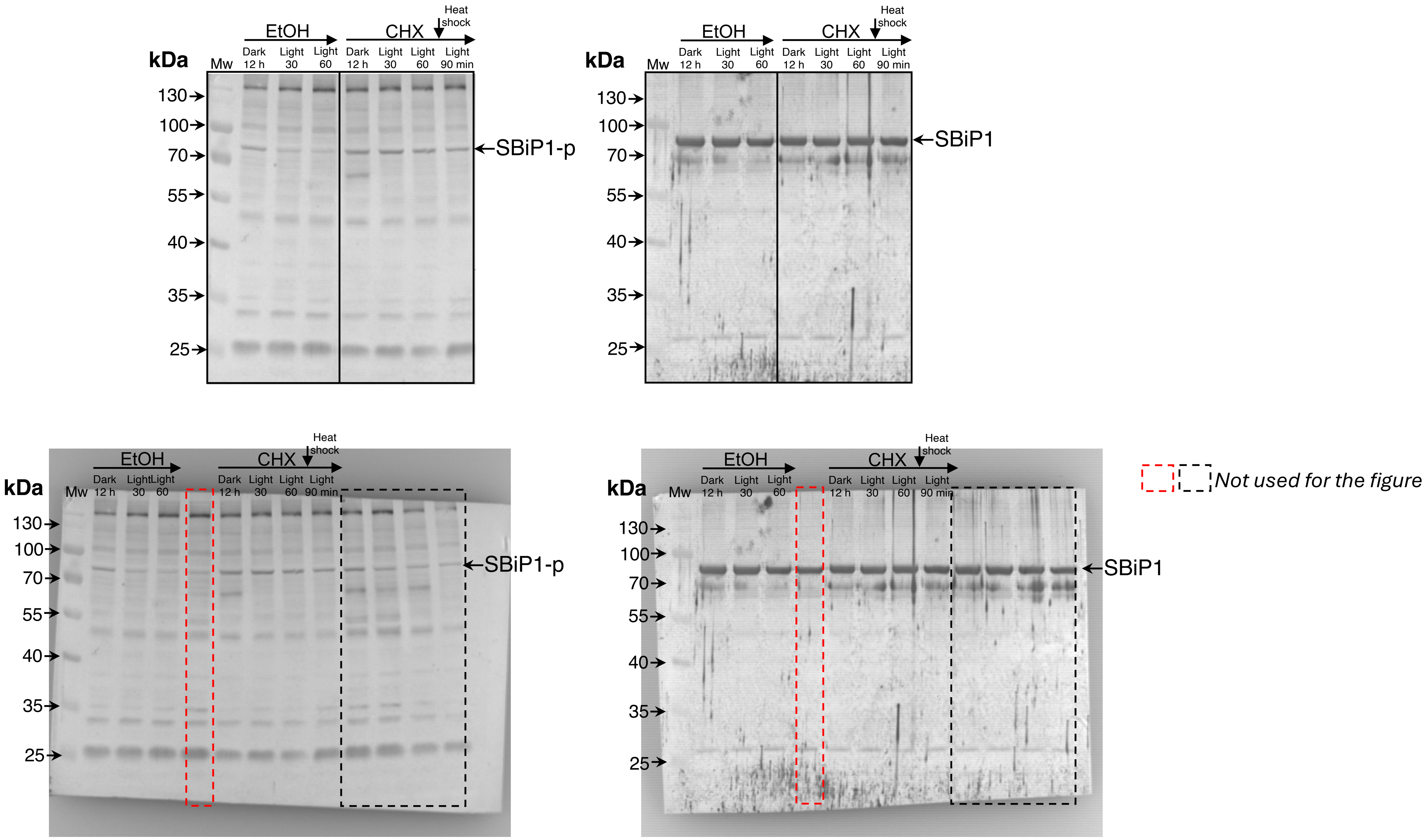

kDa
Mw
EtOH
CHX
Heat shock
Dark 12 h
Light 30
Light 60
Light 90 min
130
100
70
55
40
35
25
SBiP1-p
SBiP1
Not used for the figure

Replicate 3

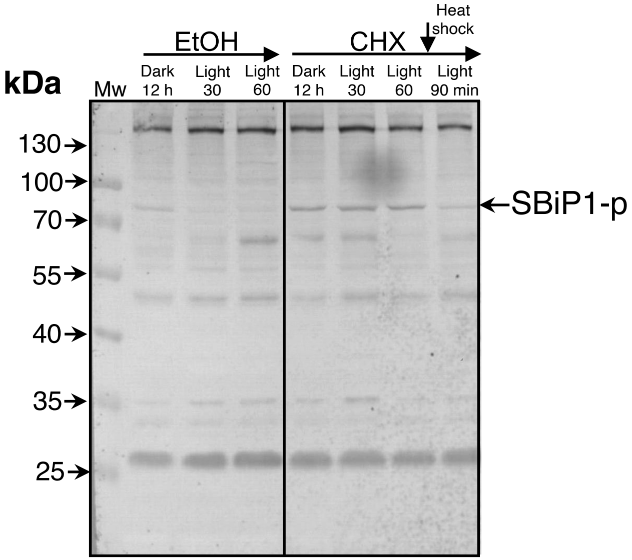

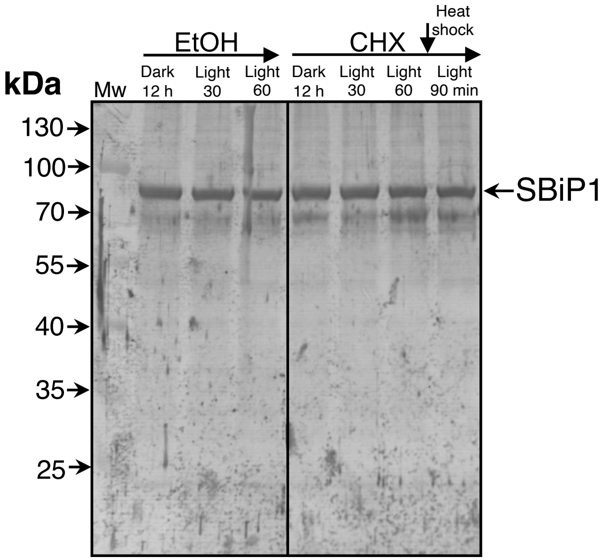

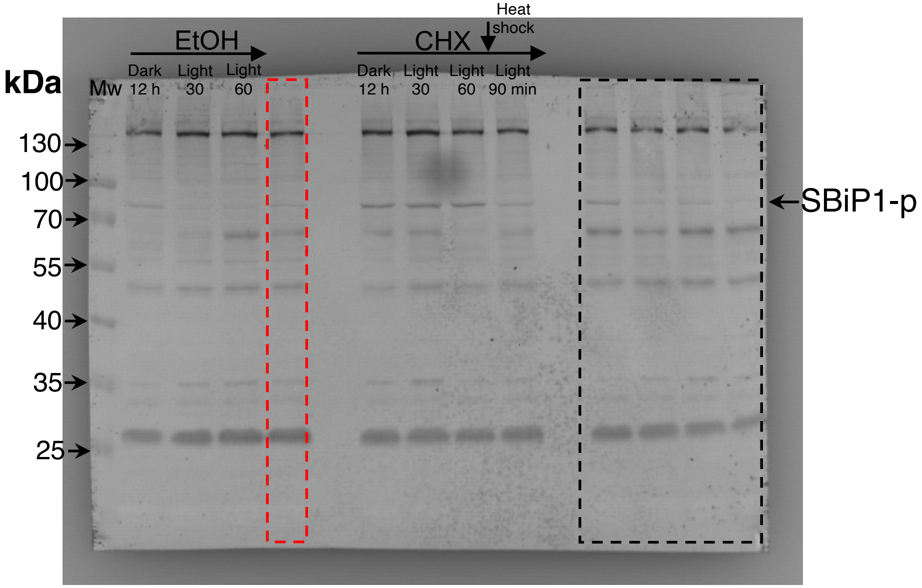

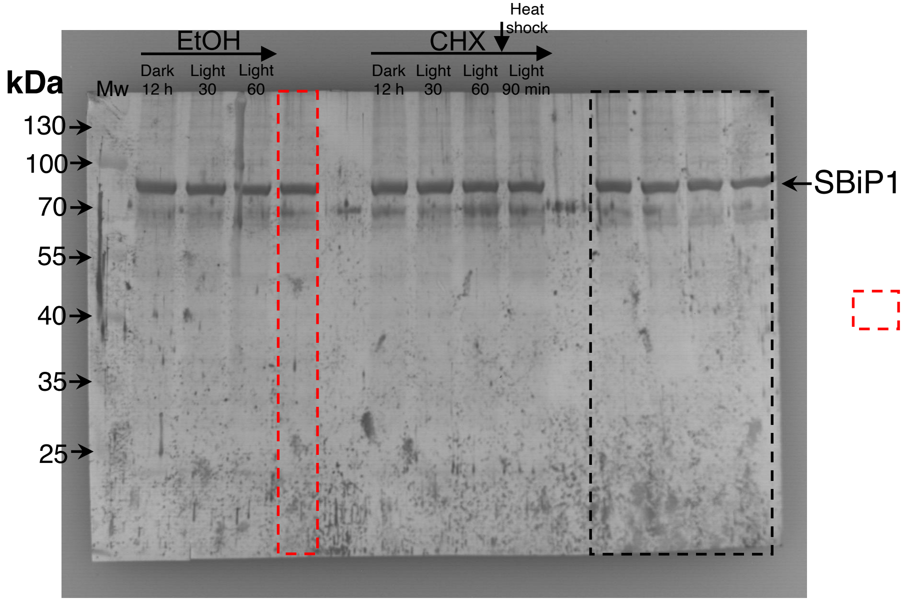

Not used for the figure

Replicate 4

Membranes from experimental replicates for Figure 4

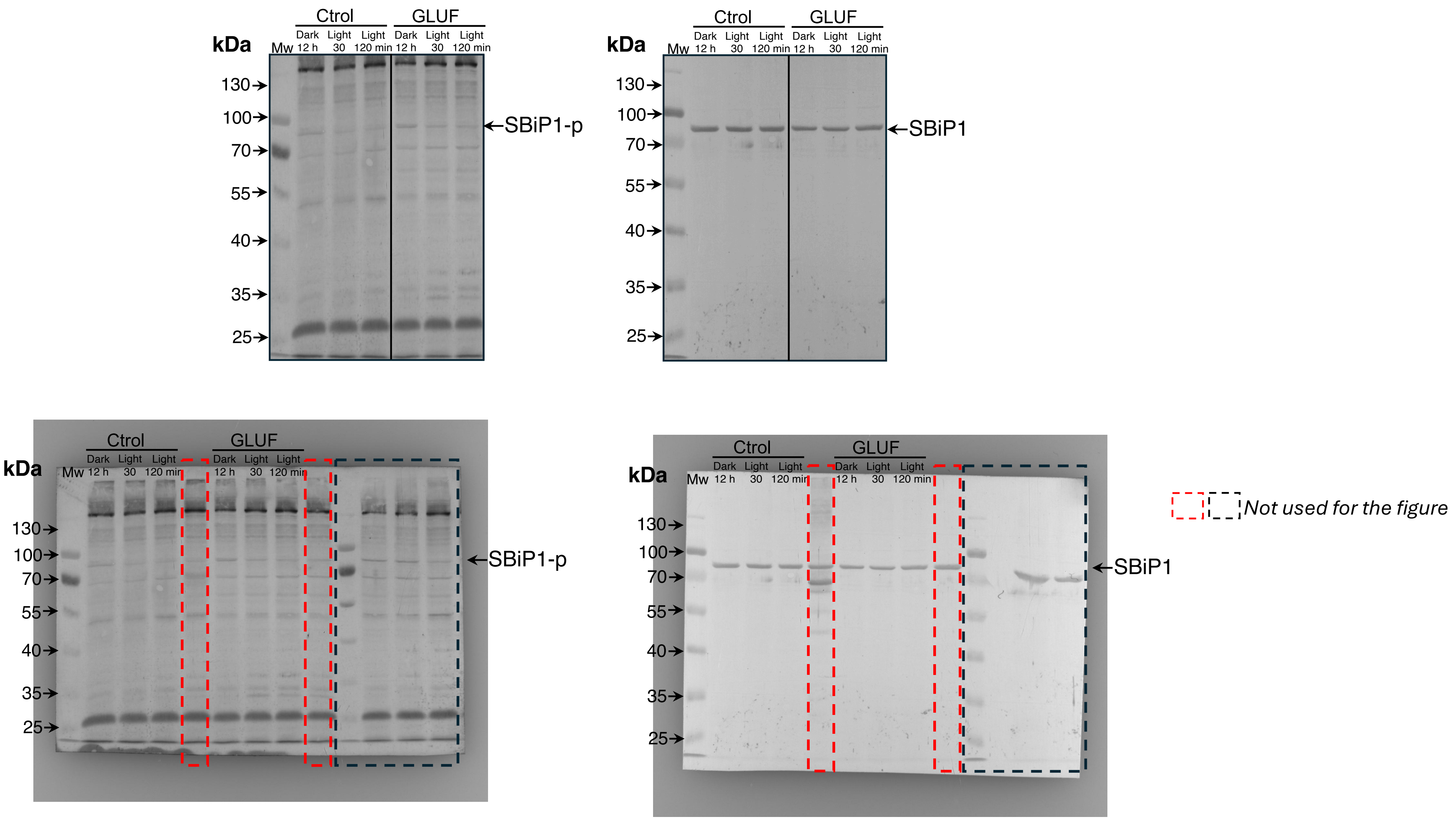

Replicate 1

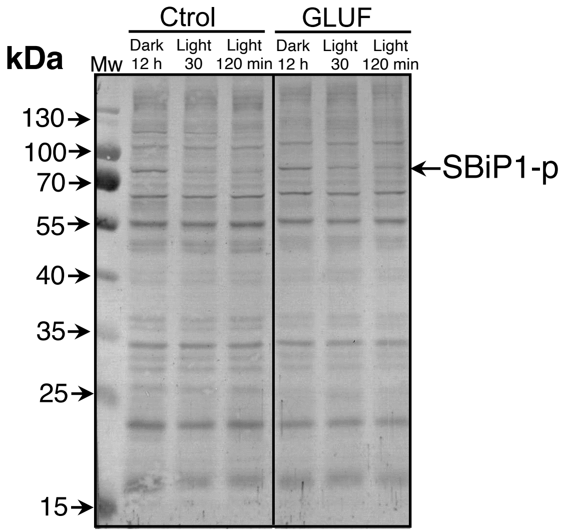

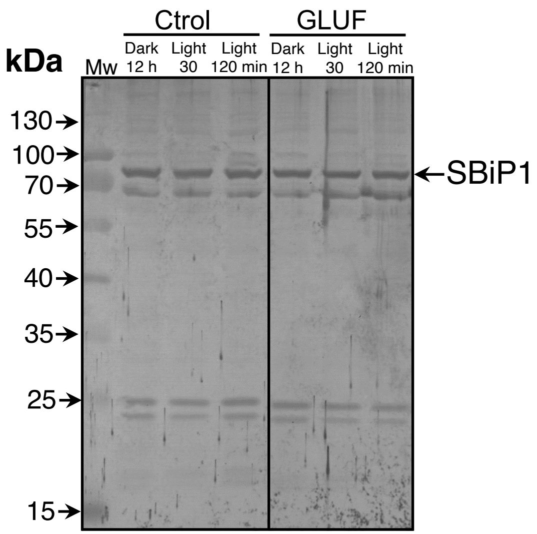

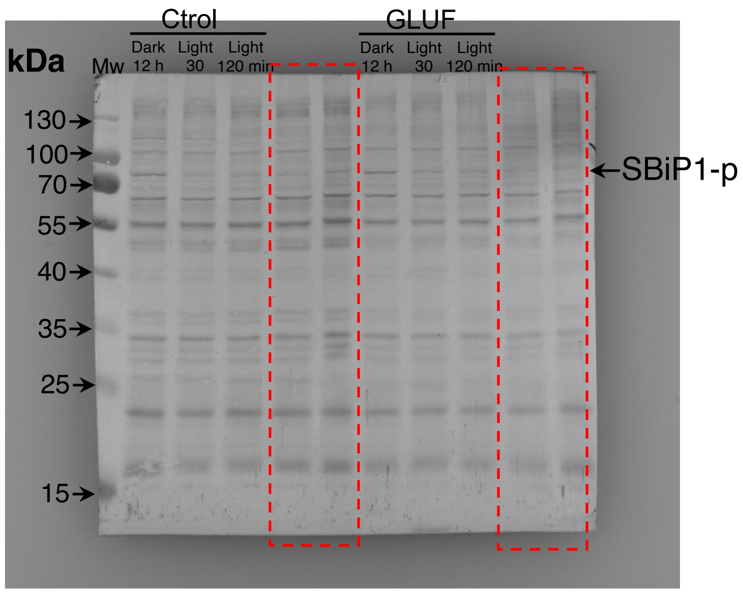

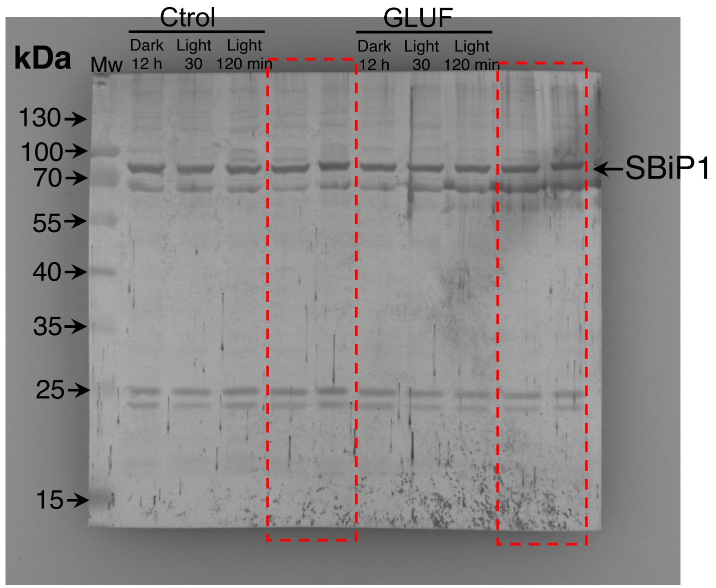

Not used for the figure

Replicate 2

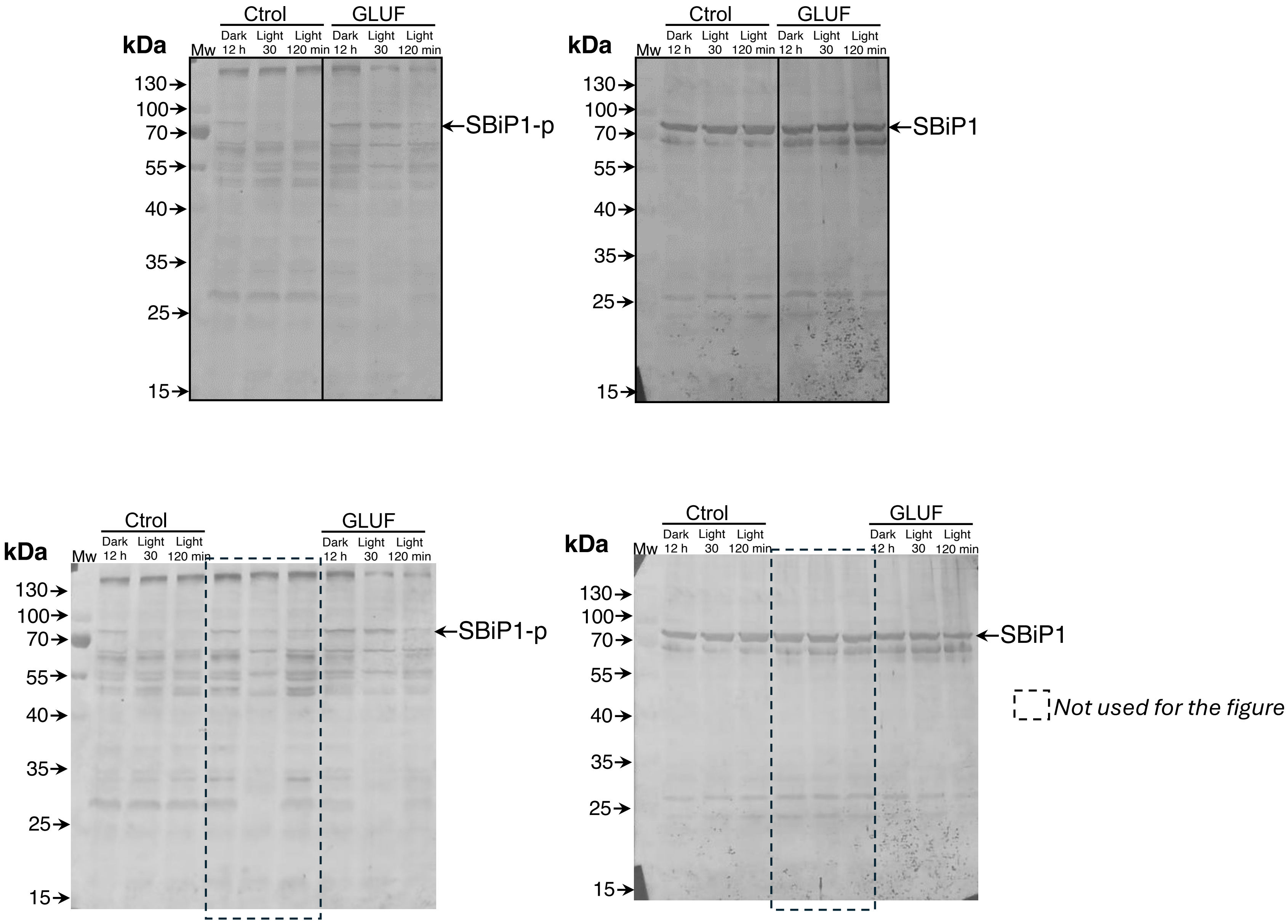
kDa
Mw
Ctrol
GLUF
Dark 12 h
Light 30
Light 120 min
130
100
70
55
40
35
25
15
SBiP1-p
SBiP1
Not used for the figure

Replicate 3

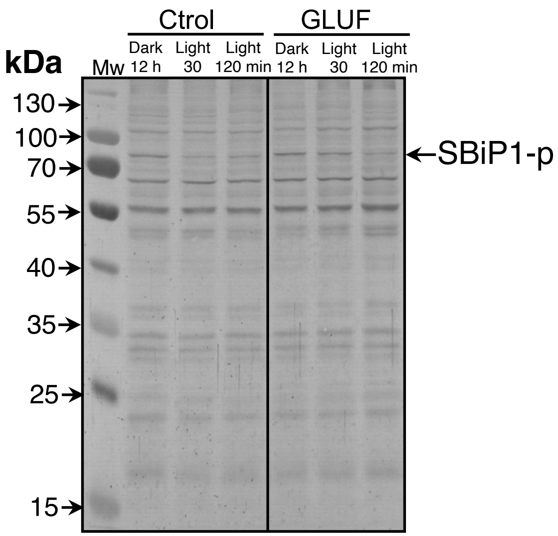

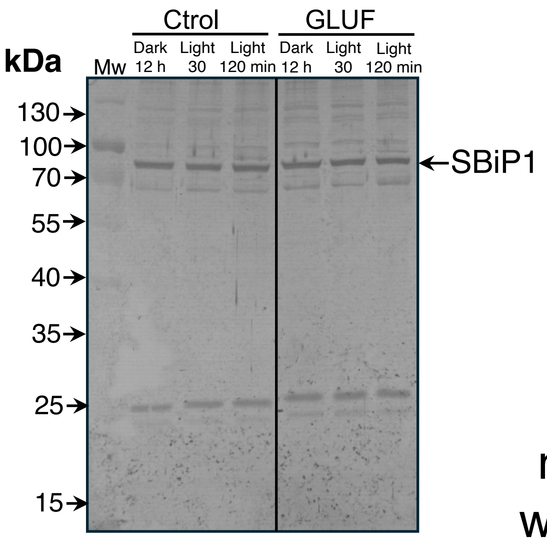

These membranes were used for Figure 4

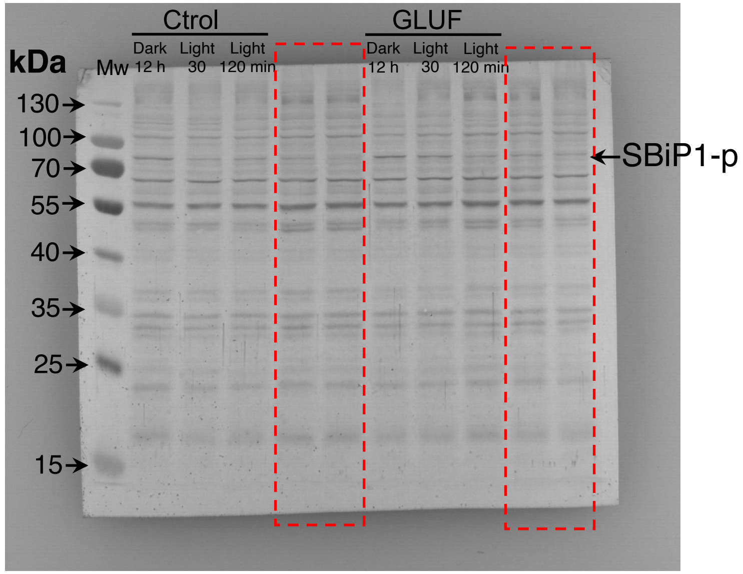

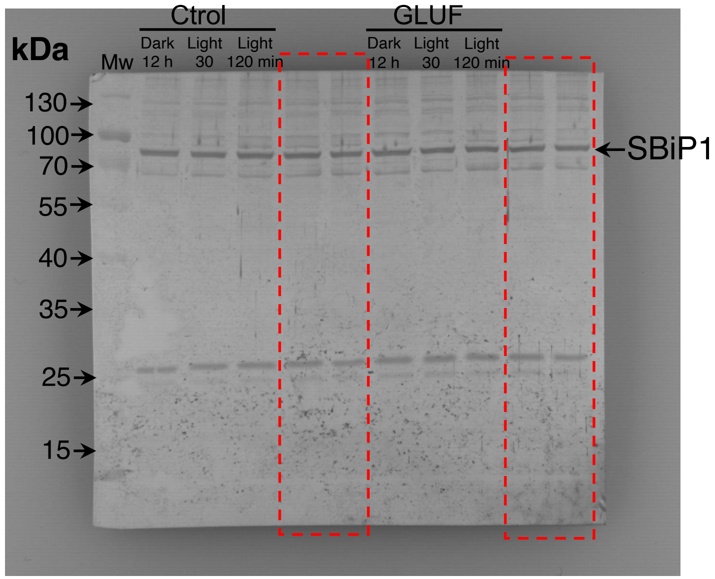

Not used for the figure

Replicate 4

Membrane for Supplementary Figure 1

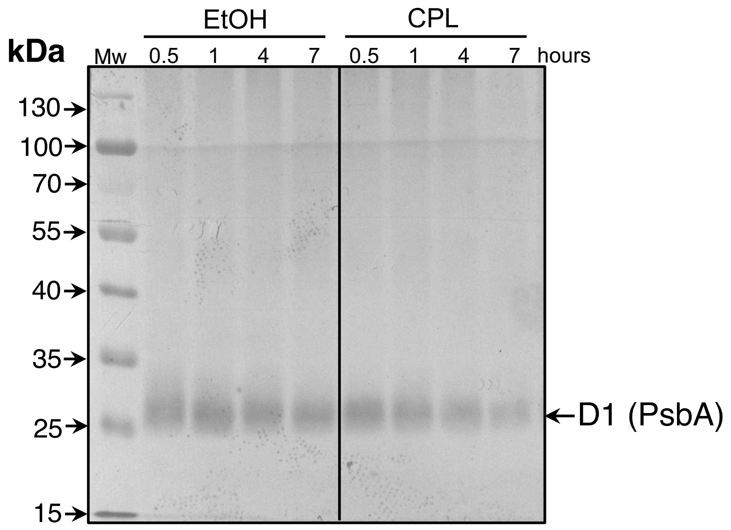

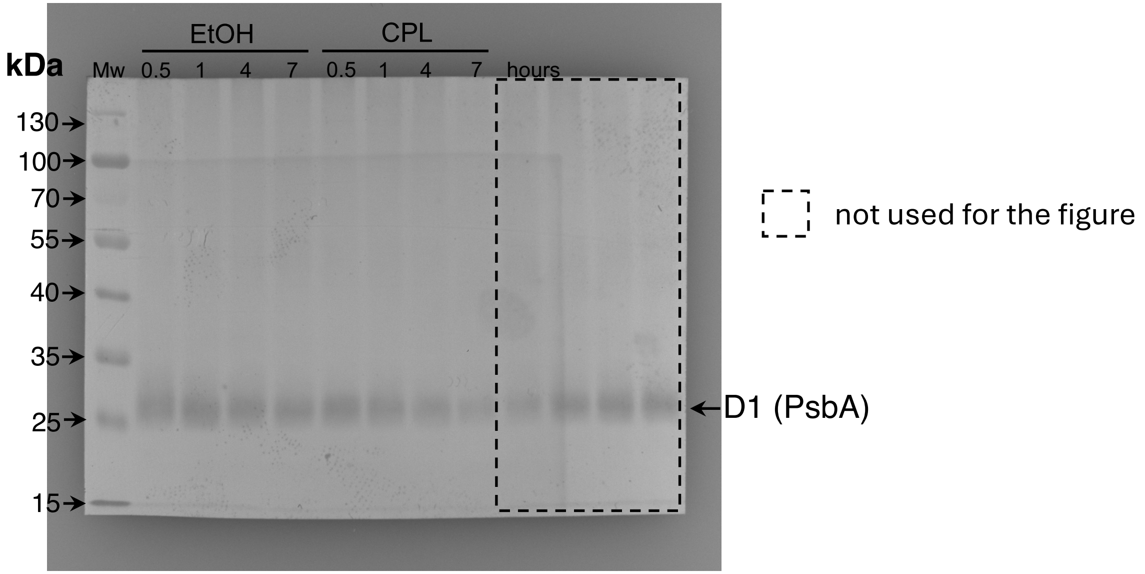

Supplement: Online supplementary material S1 [file BSR-45-06-BSR20241085-s002.pdf]
